# Supplementary material for: Comparative and functional genomic analysis of foreign DNA defense mechanisms in Enterococcus faecium
Source: Microbiol Spectr. 2025 Jun 18;13(8):e00289-25. doi: 10.1128/spectrum.00289-25 (PMC12323599; doi:10.1128/spectrum.00289-25)
Supplement: Supplemental material — Fig. S1 to S5; Tables S2, S4, S5, and S7. [file spectrum.00289-25-s0001.pdf]

Supplementary Figures and Tables:

Comparative and functional genomic analysis of foreign DNA defence mechanisms in *Enterococcus faecium*

Alexandra L. Krause[1], Louise M. Judd[1,2], Ryan Wick[1,2], Timothy P. Stinear[1,2], Andrew H. Buultjens[1] and Ian R. Monk[1].

Table of contents:

Figures

- Supplementary Figure 1: Protein alignment of the four dominant variants of the HsdMSR systems. **(PAGE 2).**
- Supplementary Figure 2: Nucleotide alignment of the HsdS_3 allele HsdMSR region in AUS0233, 2394 and 2397. **(PAGE 3).**
- Supplementary Figure 3: Alignment of the plasmid associated HsdMSR on the <200kb plasmid in strains 2405 and 2406. **(PAGE 4).**
- Supplementary Figure 4: Protein homology of the 14 HsdS alleles. **(PAGE 5).**
- Supplementary Figure 5: Protein alignment and presence of four ArdA alleles. **(PAGE 6).**

Tables

- Supplementary Table 1. Global *E. faecium* metadata from the 805 strains. See .xls.
- Supplementary Table 2. Letter code for the 20 test panel strains labelled on Figure 1. **(PAGE 7).**
- Supplementary Table 3. Gene presence absence analysis of *E. faecium* strains (A) Gene presence/absence of strains 2394 and 2397. (B) Gene presence absence analysis in the <200kb plasmid of strains 2405, 2406, 2395 and 2396. See .xls.
- Supplementary Table 4: HsdS allele, predicted recognition motif and protein accession number. **(PAGE 8).**
- Supplementary Table 5: Global presence of HsdS subunits and Type IIG systems in 32 countries across 805 *E. faecium* strains. **(PAGE 9-10).**
- Supplementary Table 6: HsdS and ArdA allele carriage across the different MLST types. See .xls.
- Supplementary Table 7. HsdS carriage across different MLST types from the 805 *E. faecium* strains. **(PAGE 10-13).**

- References. **(PAGE 13).**

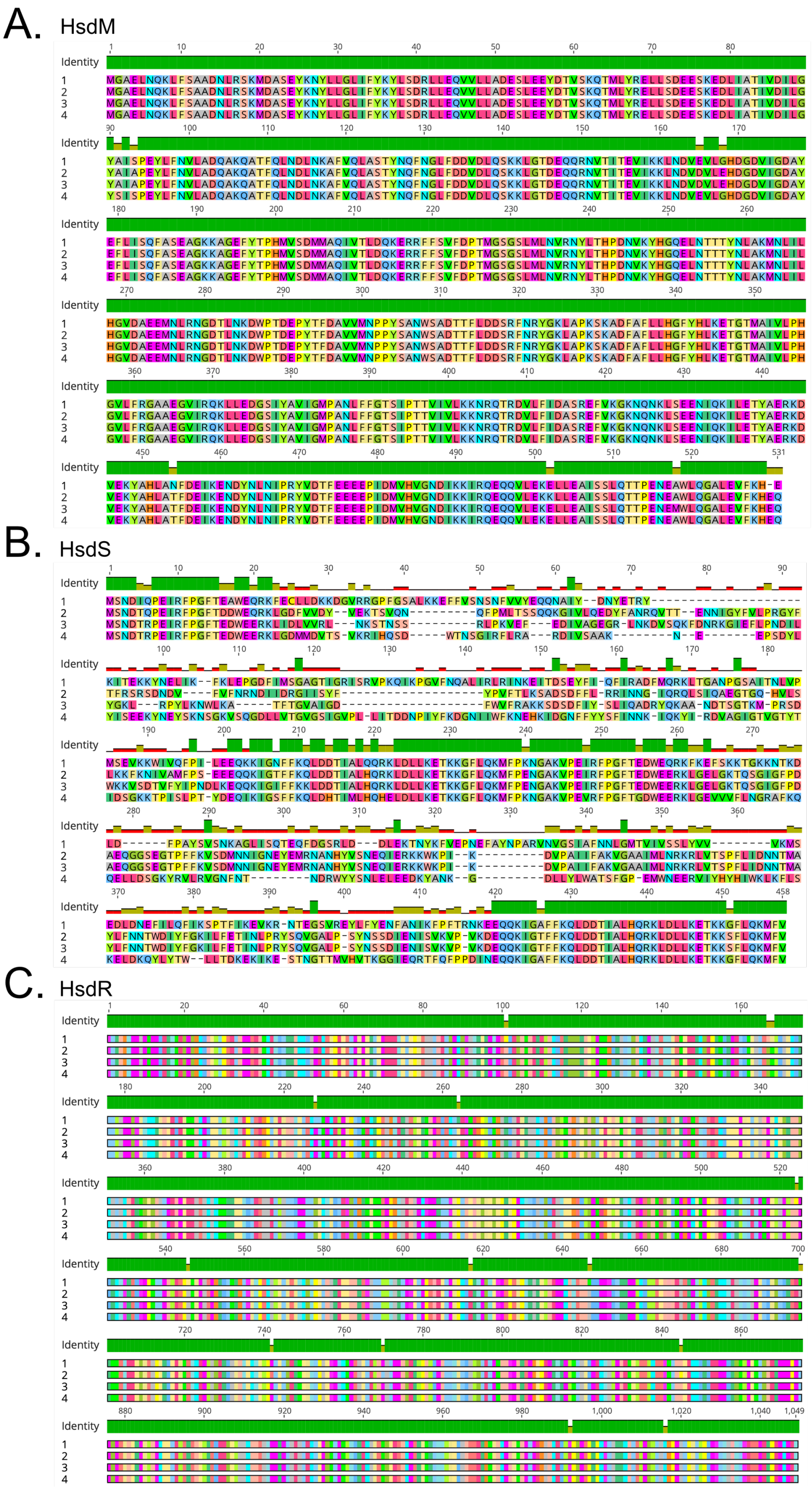

Supplementary Figure 1. Protein alignment (Clustal Omega) of the four dominant variants of the HsdRMS system (HsdS alleles: labelled 1 to 4) present in the panel of twenty *E. faecium* isolates. **(A)** HsdM, **(B)** HsdS and **(C)** HsdR.

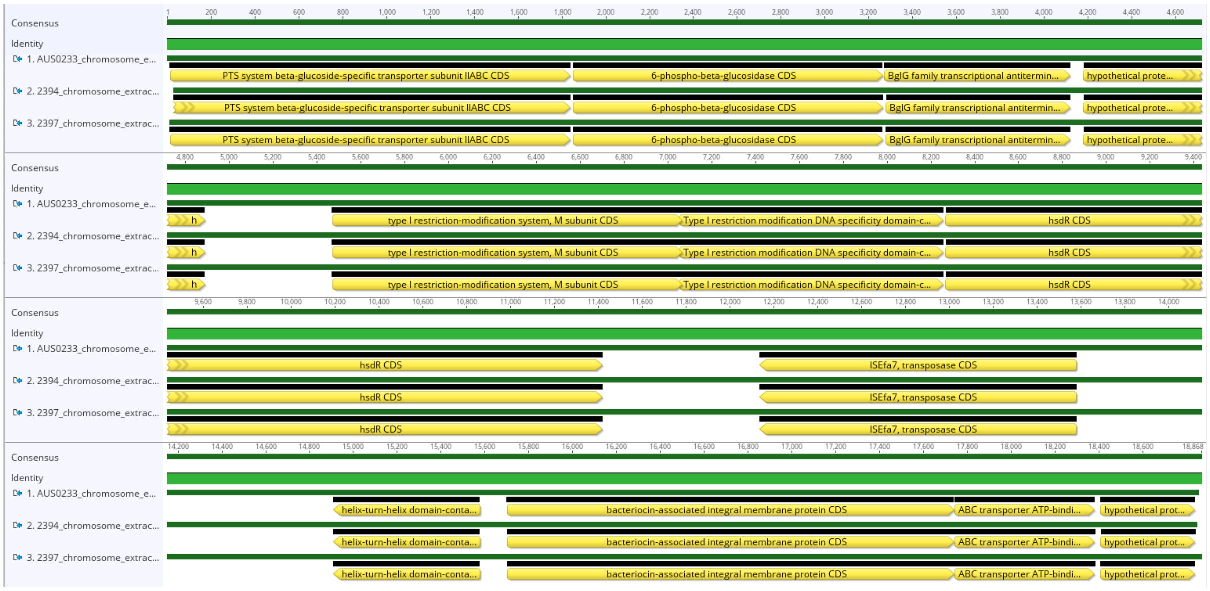

Supplementary Figure 2. Nucleotide alignment (Clustal Omega) from strains 2394, 2397 and AUS0233 in the region encompassing the HsdMSR system (HsdS_3 allele).

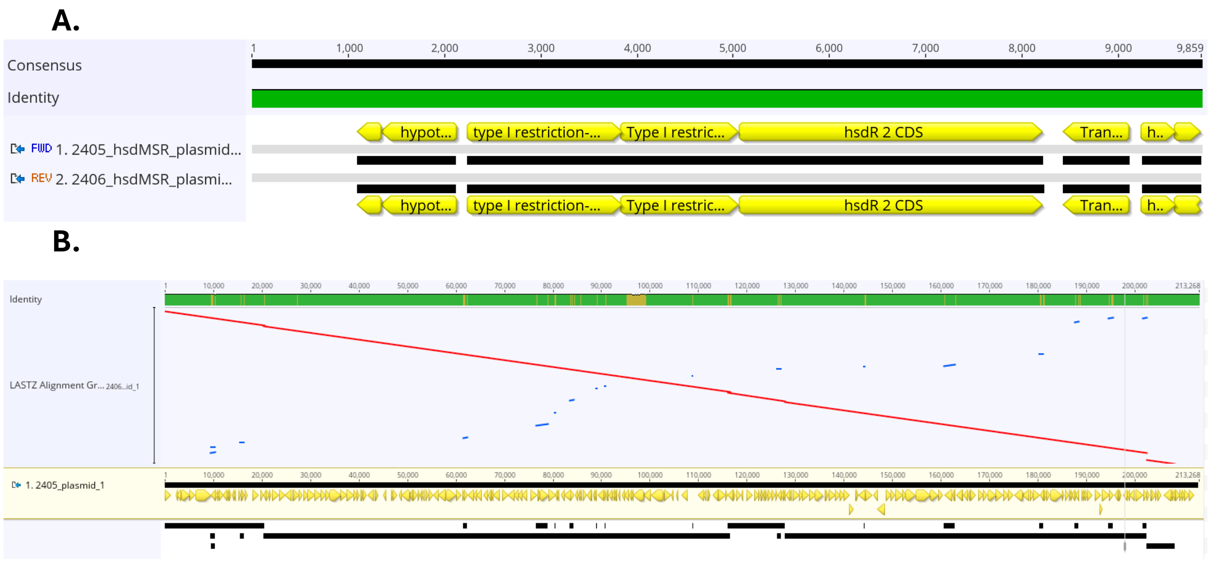

Supplementary Figure 3. Alignment of the HsdMSR on the <200kb plasmid in strains 2405 and 2406. **(A)** Nucleotide alignment (Clustal Omega) of the region surrounding the HsdMSR locus (HsdS_4 allele) in strains 2405 and 2406. **(B)** LASTZ nucleotide alignment of the <200kb plasmid containing the HsdS_4 allele in strains 2405 and 2506 (1).

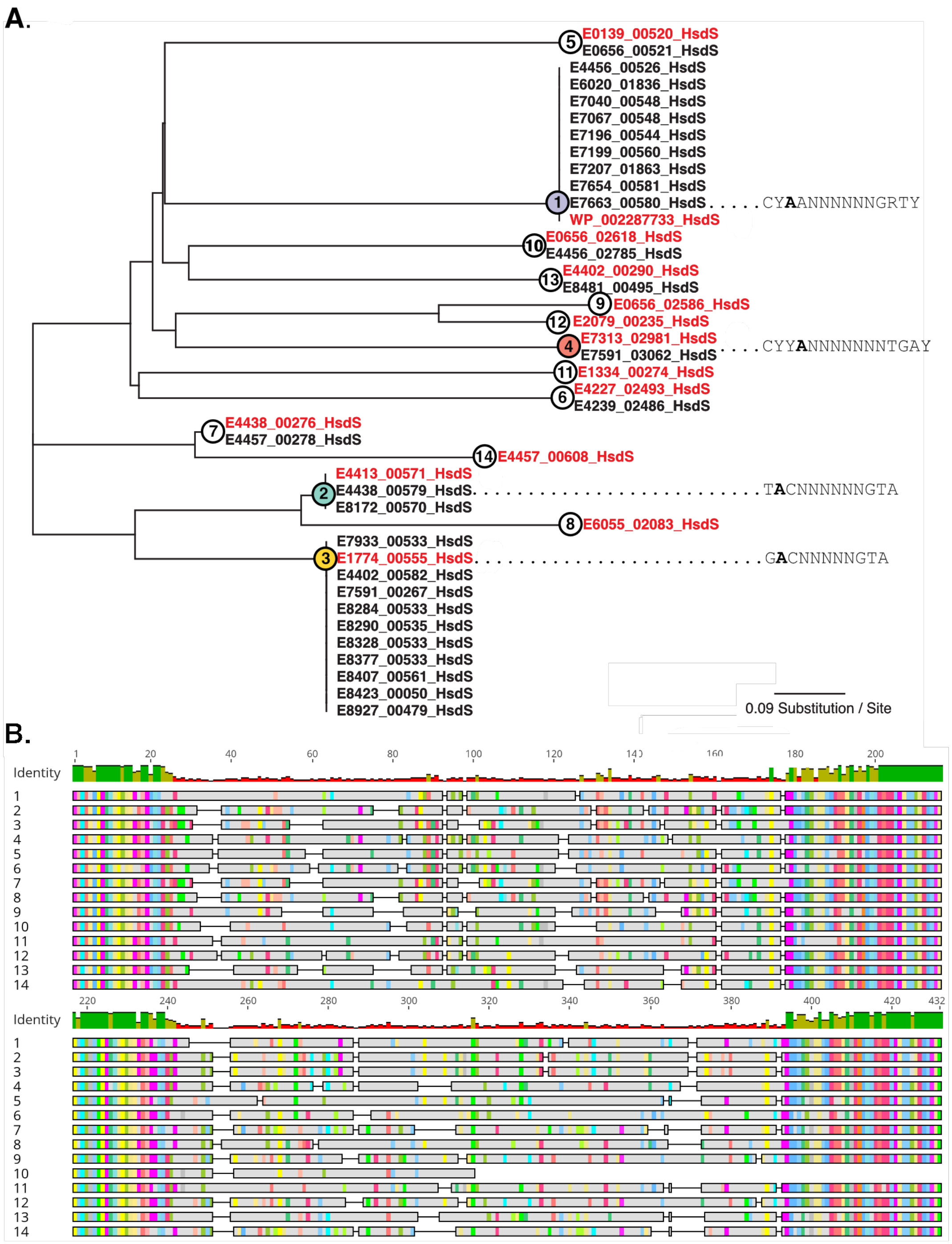

Supplementary Figure 4. Protein homology of the 14 HsdS alleles. (A) Protein phylogeny of the HsdS amino acid sequences described in this study and previously (2). Red signifies the representative HsdS allele (see Supplementary Table 2 for the protein accession). The number at the end of the node denotes the HsdS allele, with HsdS_1 to 4 colored to match colors used the main text. Where known the HsdS recognition motif is included **(B)** Amino acid alignment (Clustal Omega) of the 14 HsdS alleles identified above.

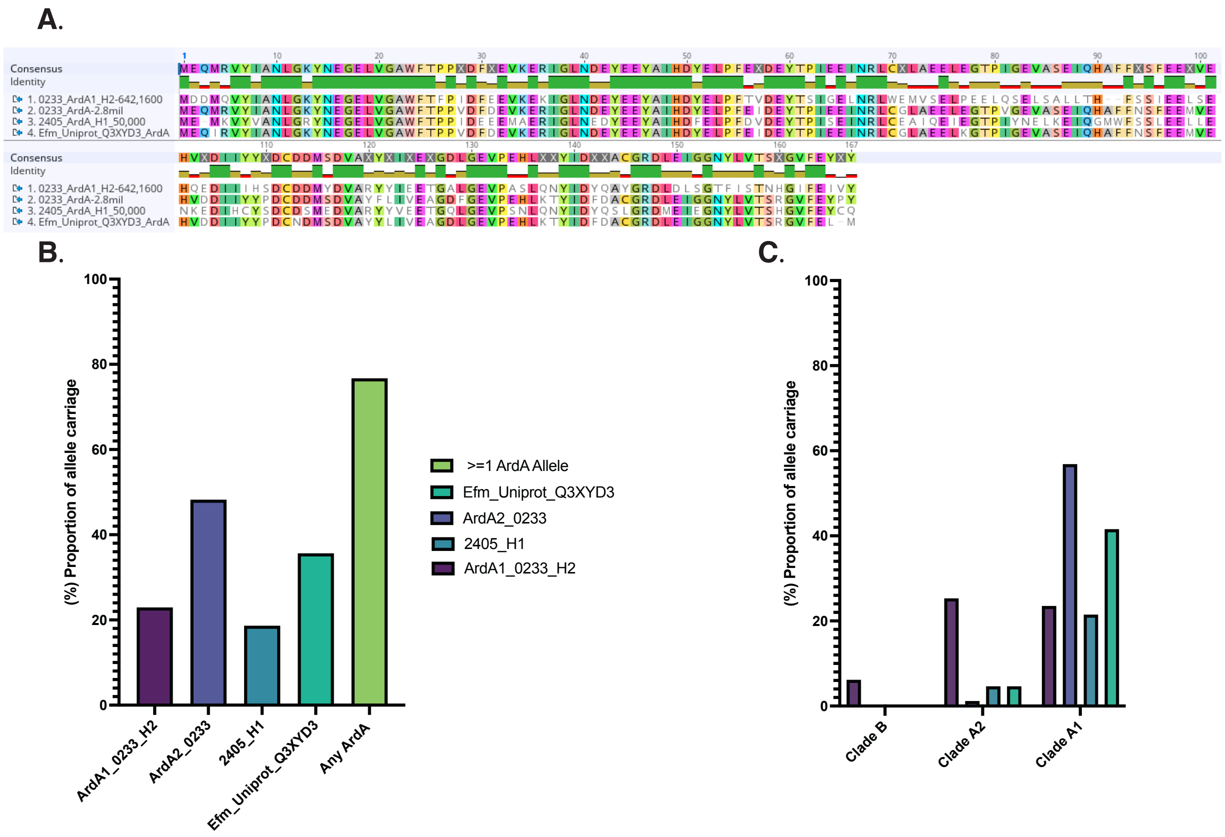

Supplementary Figure 5. (A) Amino acid alignment of the four different ArdA alleles. **(B)** Proportion of ArdA allele carriage in 805 global *E. faecium* strains from BlastX search (100%ID, LEN :(ArdA1_023_H2 165aa, ArdA2_0233 167aa, 2405_H1 166aa and Efm_Uniprot_Q3XYD3 166aa)) **(C)** ArdA allele carriage in clade B, A1, and A2 from 805 global *E. faecium* strains.

Supplementary Table 2. Letter code for 20 test panel strains labelled on Figure 1.

Letter Code	Test Panel Strain ID
**A**	2389
**B**	2397
**C**	2399
**D**	2394
**E**	2393
**F**	2403
**G**	2405
**H**	2395
**I**	2396
**J**	2406
**K**	2400
**L**	2407
**M**	2402
**N**	2404
**O**	2401
**P**	AUS0233
**Q**	2398
**R**	2392
**S**	2390
**T**	2391

Supplementary Table 4. HsdS allele key, their predicted recognition motif and protein accession number.

HsdS ID	Allele	Recognition Motif	Protein Accession
WP_002287733_HsdS	**1**	CY**A**ANNNNNNGRTY	WP_002287733.1
E4413_00571_HsdS	**2**	T**A**CNNNNNNGTA	WP_002330870.1
E1774_00555_HsdS	**3**	G**A**CNNNNNGTA	WP_002347317.1
E7313_02981_HsdS	**4**	CYY**A**NNNNNNNTGAY	WP_242618483.1
E0139_00520_HsdS	**5**	unknown	WP_002332926.1
E4227_02493_HsdS	**6**	unknown	WP_002324520.1
E4438_00276_HsdS	**7**	unknown	WP_126309379.1
E6055_02083_HsdS	**8**	unknown	WP_002341679.1
E0656_02586_HsdS	**9**	unknown	WP_231435427.1
E0656_02618_HsdS	**10**	unknown	WP_238652364.1
E1334_00274_HsdS	**11**	unknown	WP_104847034.1
E2079_00235_HsdS	**12**	unknown	WP_172597225.1
E4402_00290_HsdS	**13**	unknown	WP_126347889.1
E4457_00608_HsdS	**14**	unknown	WP_127837358.1

Supplementary Table 5. Global presence of HsdS subunits and Type IIG systems from 32 countries in 805 *E. faecium* strains.

	Type I RM HsdS Subtype						
**Country of Origin**	**HsdS _1 (%)**	**HsdS _2 (%)**	**HsdS _3 (%)**	**HsdS _4 (%)**	**Type IIG (%)**	**Number of strains**	**Year of isolation**
Test Panel (Australia)	9%	35%	38%	45%	4%	20	2012-2018
Argentina	0.0%	0.3%	0.6%	0.0%	0.0%	3	1998-2000
Australia	25.7%	12.4%	22.8%	11.6%	0.4%	241	1997-2015
Belgium	27.3%	9.1%	9.1%	9.1%	0.0%	11	1995-2011
Brazil	77.7%	0.0%	22.2%	22.2%	0.0%	9	1997-2001
Canada	0.0%	0.0%	100.0%	0.0%	0.0%	1	2001
Chile	0.0%	0.0%	100.0%	0.0%	0.0%	2	2003-2004
Denmark	29.9%	4.5%	22.4%	16.4%	0.0%	67	1995-2015
Finland	0.0%	0.0%	100.0%	0.0%	0.0%	2	1996
France	38.5%	15.4%	7.7%	0.0%	0.0%	13	1986-2000
Germany	36.1%	9.0%	5.7%	8.2%	0.0%	122	1995-2016
Hungary	100.0%	0.0%	0.0%	0.0%	0.0%	1	2005
India	100.0%	100.0%	0.0%	0.0%	0.0%	1	2015
Ireland	0.0%	0.0%	0.0%	0.0%	0.0%	1	2001
Israel	0.0%	50.0%	0.0%	0.0%	0.0%	2	1997
Italy	20.0%	0.0%	40.0%	20.0%	0.0%	5	1999-2010
Japan	21.9%	9.4%	22.9%	8.3%	0.0%	96	2014-2015
Latvia	0.0%	100.0%	0.0%	0.0%	0.0%	1	2010
Netherlands	46.4%	17.9%	10.7%	0.0%	0.0%	28	1957-2013
Norway	38.5%	0.0%	0.0%	23.1%	0.0%	13	1956-2015
Paraguay	0.0%	0.0%	100.0%	0.0%	0.0%	2	2005-2006
Poland	0.0%	0.0%	50.0%	0.0%	0.0%	2	2005
Portugal	0.0%	50.0%	50.0%	0.0%	0.0%	6	1999-2010
Saudi Arabia	0.0%	0.0%	100.0%	0.0%	0.0%	3	2000
Singapore	0.0%	0.0%	100.0%	0.0%	0.0%	1	2004
South Africa	100.0%	0.0%	0.0%	0.0%	0.0%	1	2001

Spain	23.7%	11.9%	33.9%	8.5%	0.0%	59	1995-2012
Sweden	0.0%	0.0%	100.0%	0.0%	0.0%	1	2004
Switzerland	0.0%	0.0%	0.0%	0.0%	0.0%	4	1996
Tanzania	87.5%	0.0%	0.0%	0.0%	0.0%	8	2001-2013
Tunisia	0.0%	0.0%	100.0%	0.0%	0.0%	1	2003
UK	32.2%	19.6%	37.5%	12.5%	0.0%	56	1992-2012
USA	26.9%	7.7%	15.4%	3.8%	0.0%	26	1991-2004

Supplementary Table 7. HsdS carriage across different MLST types from the 805 *E. faecium* strains.

MLST type	HsdS_1 to 14	Associated Clade - hBAPs5
21	7	**B/A2**
26	2	**B/A2/A1**
60		B
74		B
85		B
92	2	B
94		**B/A2**
104	2	B
159		B
160		B

178		B
310	6	B
361	3	B
583		B
623		B
816		B
928		B
5	5	A2
9	6	A2
12		A2
22		**A2/A1/B**
27		A2
32		A2
55		A2
57		A2
61		A2
66		**A2/A1**
69		A2
70		A2
75		A2
77		A2
102		A2
110		A2
112		A2
113		A2
115		A2
127		A2
150		A2
151		A2
158		A2
163		A2
170	3	A2
210	8	A2
286		A2
289		A2
296		**A2/B**
327		A2
328		A2
358		A2
413	2	A2
533		A2
639		A2
640		A2
709		A2
867		A2
872	2	A2
888		A2
929		A2

1175		A2
6	5	A1
16	1	A1
17	1, 2,3, 5	A1
18	1, 2,3,8	A1
20	1	A1
22	2	A1
25		A1
32		A1
50	2	A1
64	1, 3	A1
66		A1
78	1, 2, 3, 4,	A1
79		A1
80	1, 2, 3, 4	A1
87		A1
88		A1
117	1, 4	A1
121		A1
123		A1
125	14	A1
132	1, 2, 8	A1
171	2	A1
173	14	A1
186	14	A1
192	1, 2, 3, 4, 8	A1
202	1, 2,	A1
203	1, 2, 3, 4,	A1
204	1	A1
208	1	A1
209		A1
233	1, 3, 4	A1
252	1, 3	A1
262	1, 4	A1
280	3	A1
290	1	A1
323	1, 2	A1
324		A1
341	1, 3, 4,	A1
375	1	A1
376	3	A1
389	2	A1
400	1	A1
412	1, 3	A1
414	1, 3,	A1
438		A1
555	2, 3	A1
664	3	A1

736	3	A1
779		A1
780		A1
796	3, 4	A1
927	1, 4	A1
958		A1
1005	1	A1
1043		A1
1196		A1
1200		A1
1201		A1
1449		A1
1483	1	A1
1484		A1
1486	1	A1
1495	4	A1
1421	1, 2, 3, 4	A1

References:

1. Harris RS. 2007. Improved pairwise alignment of genomic DNA. The Pennsylvania State University.
2. Arredondo-Alonso S, Top J, McNally A, Puranen S, Pesonen M, Pensar J, Marttinen P, Braat JC, Rogers MRC, van Schaik W, Kaski S, Willems RJL, Corander J, Schurch AC. 2020. Plasmids Shaped the Recent Emergence of the Major Nosocomial Pathogen *Enterococcus faecium*. mBio 11.
